# Application of total keratometry in ten intraocular lens power calculation formulas in highly myopic eyes

**DOI:** 10.1186/s40662-022-00293-3

**Published:** 2022-06-09

**Authors:** Ling Wei, Kaiwen Cheng, Wenwen He, Xiangjia Zhu, Yi Lu

**Affiliations:** 1 Department of Ophthalmology and Eye Institute, Eye and ENT Hospital of Fudan University, Shanghai, 200031 China; 2 Key Laboratory of Myopia (Fudan University)Key Laboratory of MyopiaShanghai Key Laboratory of Visual Impairment and Restoration, National Health Commission, Chinese Academy of Medical Science, Shanghai, 200031 China; 3 State Key Laboratory of Medical Neurobiology, Fudan University, Shanghai, 200031 China

**Keywords:** High myopia, Total keratometry, Cataract surgery, IOL power calculation

## Abstract

**Background:**

The accuracy of using total keratometry (TK) value in recent IOL power calculation formulas in highly myopic eyes remained unknown.

**Methods:**

Highly myopic patients who underwent uneventful cataract surgery were prospectively enrolled in this prospective comparative study. At one month postoperatively, standard deviation (SD) of the prediction errors (PEs), mean and median absolute error (MedAE) of 103 highly myopic eyes were back-calculated and compared among ten formulas, including XGboost, RBF 3.0, Kane, Barrett Universal II, Emmetropia Verifying Optical 2.0, Cooke K6, Haigis, SRK/T, and Wang-Koch modifications of Haigis and SRK/T formulas, using either TK or standard keratometry (K) value.

**Results:**

In highly myopic eyes, despite good agreement between TK and K (*P* > 0.05), larger differences between the two were associated with smaller central corneal thickness (*P* < 0.05). As to the refractive errors, TK method showed no differences compared to K method. The XGBoost, RBF 3.0 and Kane ranked top three when considering SDs of PEs. Using TK value, the XGboost calculator was comparable with the RBF 3.0 formula (*P* > 0.05), which both presented smaller MedAEs than others (all *P* < 0.05). As for the percentage of eyes within ± 0.50 D or ± 0.75 D of PE, the XGBoost TK showed comparable percentages with the RBF 3.0 TK formula (74.76% vs. 66.99%, or 90.29% vs. 87.38%, *P* > 0.05), and statistically larger percentages than the other eight formulas (*P* < 0.05).

**Conclusions:**

Highly myopic eyes with thinner corneas tend to have larger differences between TK and K. The XGboost enhancement calculator and RBF 3.0 formula using TK showed the most promising outcomes in highly myopic eyes.

**Supplementary Information:**

The online version contains supplementary material available at 10.1186/s40662-022-00293-3.

## Background

Nowadays, accurate refractive predictions are of great importance to refractive cataract procedures. Apart from improved surgical techniques, modern biometry and new generation of formulas are two other crucial determinants. Despite the promising outcomes reached in the intraocular lens (IOL) power calculation of non-myopes [1], making accurate IOL power predictions for highly myopic eyes remains quite challenging. It is especially difficult due to unreliable corneal power measurements of highly myopic eyes [2–5], and compounding effects of other scenarios, including previous refractive surgeries [6].

Accurate assessment of the corneal curvature is essential for IOL power prediction. Conventionally, the corneal power was estimated with a theoretical algorithm, using anterior corneal curvature only [7]. Now, total keratometry (TK) can be calculated using the newest IOLMaster 700, which measures anterior and posterior corneal curvatures, as well as corneal thickness. By replacing assumptions with actual measurements, the application of TK may provide additional benefits for IOL power calculations.

The TK might also improve the refractive prediction in highly myopic eyes. Traditional IOL power calculation formulas based on normal eyes might generate unexpected errors for highly myopic eyes [8]. Two vergence formulas, the SRK/T and Haigis, have shown acceptable outcomes in highly myopic eyes after Wang-Koch (WK) modifications for axial length (AL) [9]. Late generation formulas, represented by the Barrett Universal II (BUII) and Olsen formulas, were found to be more promising in long eyes [10]. More recently, formulas based on artificial intelligence (AI), such as the Kane, Radial Basis Function (RBF 3.0), and the XGBoost enhancement calculator we developed, have further improved outcomes [11–14]. Accuracies of other new formulas such as Cooke K6 formula, and Emmetropia Verifying Optical (EVO 2.0) formulas, remained unknown in highly myopic eyes. Rare previous studies have compared the performance of IOL calculation formulas when applying the new TK over standard keratometry (K) values in normal AL eyes [15, 16], or in all AL ranges [13, 17], but few in long AL eyes.

In this study, we hypothesized that TK may also fit well with current new formulas when dealing with highly myopic eyes and can somehow differ from the standard K method. Therefore, we conducted a prospective study to investigate the performance of ten IOL power calculation formulas, including the XGBoost, RBF 3.0, Kane, BUII, K6, EVO 2.0, Haigis, SRK/T, and WK modifications of Haigis and SRK/T formulas, using the new TK method in highly myopic eyes.

## Methods

### Ethics

In adherence with the Declaration of Helsinki and its amendments, this prospective comparative study was conducted with approval from the Ethics Committee of Eye and ENT Hospital of Fudan University (Shanghai, China, No. 2013021). Signed informed consent was obtained from all patients, who were informed of the usage of their clinical data. This study was affiliated with the Shanghai High Myopia Study, which was registered at www.clinicaltrials.gov (accession number NCT03062085).

### Patients and eligibility

Patients who underwent uneventful cataract surgeries and IOL implantations in our hospital from December 2020 to August 2021 were enrolled in this one-year prospective study. The inclusion criteria were eyes with (1) AL ≥ 26.00 mm [10, 18, 19]; (2) successful biometry with the IOLMaster 700, including TK method; and (3) implanted HumanOptics MC X11 ASP IOL. Patients were excluded if they had previous ocular surgeries or traumas; had lid disorders, corneal opacity, glaucoma, zonular weakness, keratoconus, uveitis or other diseases that may have influenced the accuracy of manifest refraction; required premium IOL implants (such as multifocal or Toric IOLs); had severe intraoperative or postoperative complications, such as posterior capsular rupture, endophthalmitis, retinal detachment, etc.; had postoperative best-corrected distance visual acuity (BCVA) worse than 20/40 or were lost to one-month follow-up. Ultimately, 103 highly myopic eyes from 103 patients were included and further divided into three subgroups, according to AL, as follows: high myopia control (26.0–28.0 mm, n = 46), extreme myopia 1 (28.0–30.0 mm, n = 29) [20], and extreme myopia 2 (30.0 mm or more, n = 28) [21, 22].

### Preoperative measurements

Routine preoperative examinations were performed, including BCVA (logarithm of the minimal angle of resolution [logMAR]), optical biometry (IOLMaster 700; Carl Zeiss Meditec, Jena, Germany, software version 1.80), corneal topography (Pentacam HR; Oculus Optikgeräte, Wetzlar, Germany, software version 1.22r05), optical coherence tomography (OCT) exam (Spectralis OCT; Heidelberg Engineering, Heidelberg, Germany, software version 6.12.4), and B-scan ultrasonography. Data for AL, anterior chamber depth (ACD) as measured from epithelium to anterior lens, lens thickness (LT), white-to-white distance (WTW), and central corneal thickness (CCT) were recorded. Both standard K and TK data were collected using the IOLMaster 700. An index of 1.3375 was used for standard K.

### Surgical procedure

All surgeries were performed by the same experienced surgeon (YL). Standard topical anesthesia was administered in all cases. Through a 2.6 mm temporal clear corneal incision, a 5.0–5.5 mm capsulorhexis was made followed by phacoemulsification. An IOL was then implanted into the bag. No sutures were used in any of the eyes. After surgery, all patients were prescribed topical prednisolone acetate (Allergan Pharmaceutical Ireland, Westport, Ireland) and levofloxacin (Cravit, Santen Pharmaceutical) to be instilled four times a day for two weeks; and pranoprofen eye drops (Pranopulin, Senju Pharmaceutical, Osaka, Japan) to be instilled four times a day for 4 weeks.

### Postoperative examinations

Ophthalmic examinations were carried out one month after surgery. uncorrected visual acuity (UCVA) and BCVA were recorded. Manifest refractions were performed by the same doctor (LW), with subjective methods, and presented as spherical equivalence (SE). The prediction error (PE) was defined as the actual postoperative SE minus predicted SE, which was back-calculated using the implanted IOL power. A negative PE indicates a postoperative refractive result that was more myopic than predicted by the individual formula. The A constants retrieved from the User Group for Laser Interference Biometry website (ocusoft.de/ulib/index.htm) and the lens factor of the BUII Formula provided by the APACRS website were used in the IOL power calculation. The accuracies of all formulas (XGBoost-based enhancement calculator optimized for highly myopic eyes [14], Hill-RBF 3.0 [13], Kane [http://www.iolformula.com], BUII [23], Cooke K6 [https://cookeformula.com/], EVO 2.0 [http://www.evoiolcalculator.com], Haigis [24] and SRK/T [25] formulas and those with WK modification of ALs [SRK/T^WK^ and Haigis^WK^] [9] were compared. The mean absolute error (MAE), the median absolute errors (MedAEs), and the percentages of eyes within ± 0.25 D, ± 0.50 D, ± 0.75 D, and ± 1.00 D of the PE (± 0.25 D %, ± 0.50 D %, ± 0.75 D %, and ± 1.00 D %) were calculated and compared.

### Statistical analysis

Based on ± 0.25 D %, a sample size of 52 eyes was needed to reach statistical significance using paired McNemar’s Chi-squared test, with an intended power of 80% and a significance level of 5% [15, 26]. Statistical analyses were performed with SPSS software (version 12.0, SPSS, Inc.). Continuous variables were described as the mean ± standard deviation (SD). The Bland-Altman method was used to visualize the agreement between the TK and K measurements. Intraclass correlation coefficient (ICC) analysis was calculated using the two-way mixed model and absolute agreement, and an ICC over 0.90 suggested high agreement and less variance between two measurements [15]. Pearson correlation analysis was applied for differences between TK and K methods and other biometric parameters, including AL, ACD, LT, WTW and CCT. One-sample student’s t test was applied to compare the mean PEs with zero in each formula. The Wilcoxon signed-rank test was used to evaluate the differences of absolute PEs between the K and TK methods. Friedman test with Bonferroni-Dunn’s post hoc correction were used to compare the absolute PEs generated by all formulas using either the TK or K method. Heteroscedastic tests for PE comparisons including F-test (for two groups) or H-test (for ≥ three groups) were applied. For ± 0.25 D %, ± 0.50 D %, ± 0.75 D %, and ± 1.00 D %, the paired McNemar’s Chi-squared test was used to compare TK and K performances, while Cochran’s Q test was used to compare the performances of all formulas. A *P* value of less than 0.05 was considered statistically significant.

## Results

### Demographic data

Demographic data and biometric parameters are presented in Table 1. The mean AL was 28.85 ± 2.34 mm, and ranged from 26.01 to 35.02 mm. The mean CCT was 546.80 ± 33.51 μm, and ranged from 474.86 to 636.41 μm.

**Table 1:** Demographic and ocular biometry data

	Mean ± SD	Range
Age (years)	64.99 ± 9.33	42 to 80
Sex (female/all)	50.49% (52/103)	–
Axial length (mm)	28.85 ± 2.34	26.01 to 35.02
Flat K (D)	42.81 ± 1.45	40.02 to 47.24
Steep K (D)	43.76 ± 1.53	40.61 to 48.06
Flat TK (D)	42.81 ± 1.46	39.87 to 47.12
Steep TK (D)	43.81 ± 1.48	40.52 to 47.97
Anterior chamber depth (mm)	3.45 ± 0.31	2.61 to 4.27
Lens thickness (mm)	4.49 ± 0.34	3.53 to 5.42
Central corneal thickness (μm)	546.80 ± 33.51	474.86 to 636.41
White-to-white distance (mm)	11.97 ± 0.39	10.94 to 13.13
Baseline BCVA (logMAR)	0.96 ± 0.63	0.22 to 3.00
Baseline cylinder (D)	− 1.02 ± 0.69	− 3.25 to 0.00
Post-op BCVA (logMAR)	0.14 ± 0.10	− 0.08 to 0.30
Post-op SE (D)	− 2.83 ± 0.90	− 5.13 to − 0.63
Implanted IOL power (D)	10.39 ± 4.64	1.00 to 18.50
Follow-up period (months)	2.43 ± 0.94	1.03 to 4.83

*K* = keratometry; *TK* = total keratometry; *D* = diopter; *BCVA* = best-corrected distant visual acuity; *logMAR* = logarithm of the minimal angle of resolution; *Post-op* = postoperative; *SE* = spherical equivalence; *IOL* = intraocular lens

### Agreement between TK and K methods and influencing factors

**Fig. 1:**
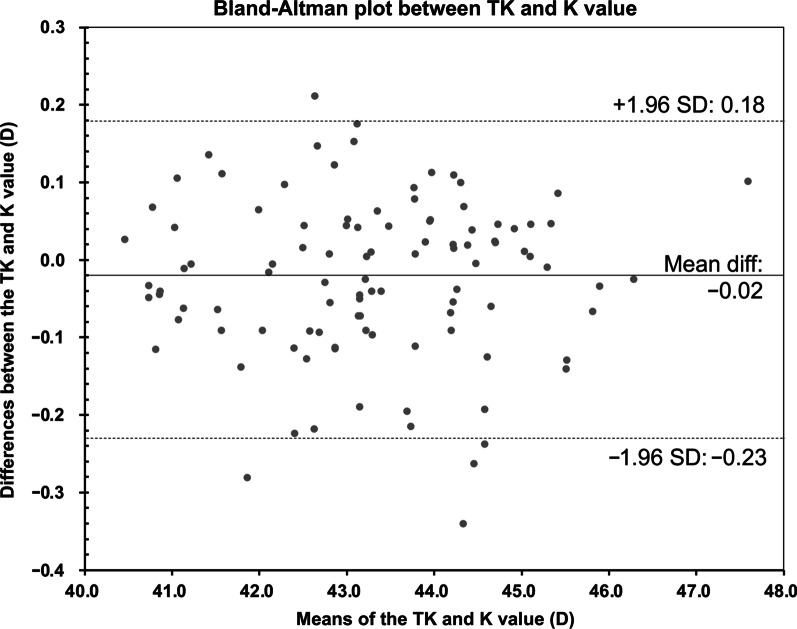
Agreement between total keratometry (TK) and standard keratometry (K) in highly myopic eyes

**Fig. 2:**
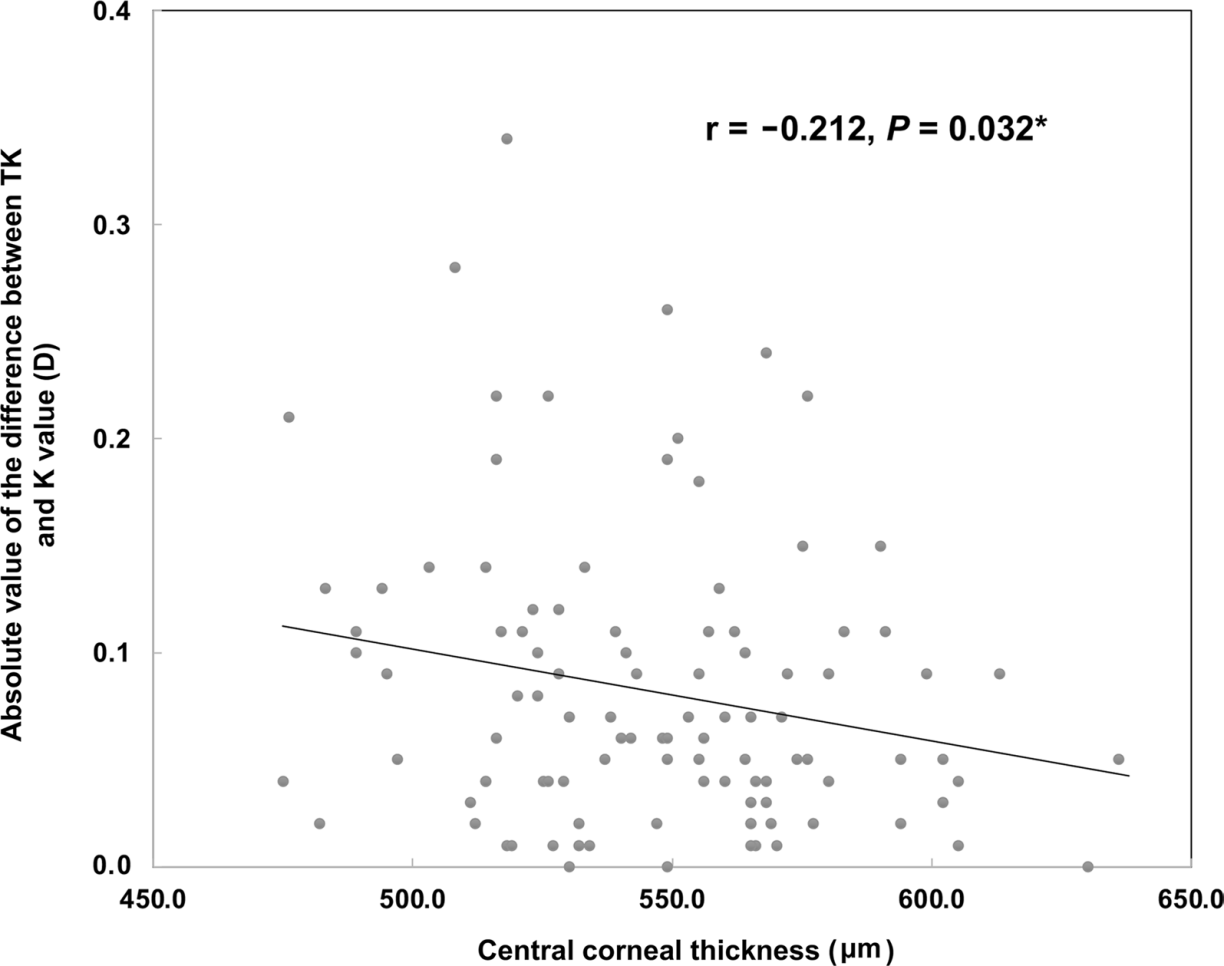
Association of the central corneal thickness and the absolute value of the difference between total keratometry (TK) and keratometry (K) methods

The agreement between TK and K methods was evaluated using the Bland-Altman test (Fig. 1). The mean difference was − 0.02 D, with 95% confidence interval (CI) range from − 0.23 to 0.18, demonstrating relatively good agreement (ICC = 0.997, *P* < 0.001). The agreements between flat TK and flat K, steep TK and steep K, and TK and K were demonstrated in Additional file 1: Table S1, all revealing high agreements (all ICCs > 0.900, *P* < 0.001). Pearson correlation analysis revealed that a larger difference between TK and K methods was associated with a thinner CCT (Fig. 2, r =  − 0.212, *P* = 0.032), but was not associated with AL, WTW, LT or the corneal radius (all *P* > 0.05).

### Prediction errors (PEs)

**Fig. 3:**
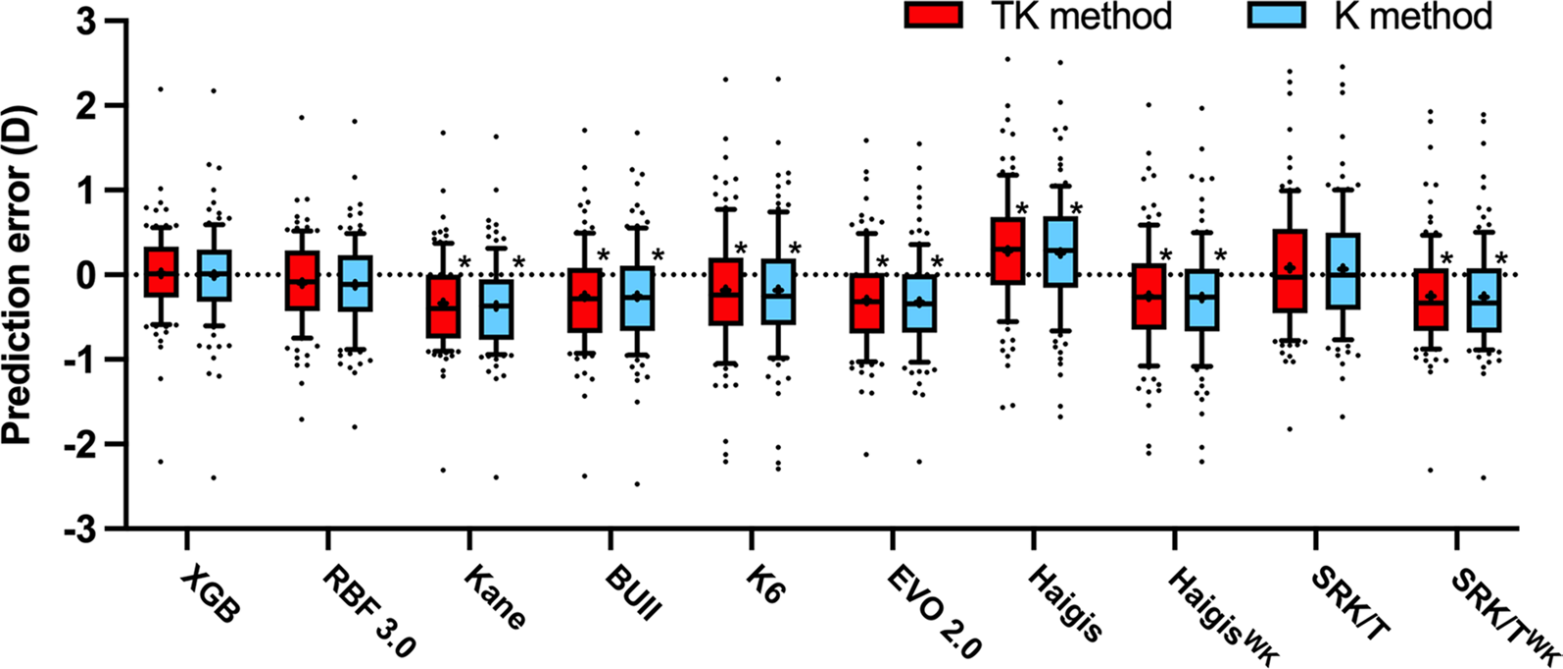
Mean prediction errors of ten formulas with total keratometry (TK) and standard keratometry (K). XGB, XGBoost enhancement calculator; RBF 3.0, Radial Basis Function formula version 3.0; BUII, Barrett Universal II formula; K6, Cooke K6 formula; EVO 2.0, Emmetropia Verifying Optical formula version 2.0. *Statistical significance (*P* < 0.05) using one-sample student’s t test to compare the mean prediction errors with zero

Mean PEs are presented in Fig. 3. The XGBoost enhancement calculator, RBF 3.0, and SRK/T formula showed emmetropic predictions (mean PE vs. 0, *P* > 0.05), the Haigis formula showed hyperopic predictions (mean PE > 0, *P* < 0.05), while other formulas showed myopic predictions (mean PE < 0, *P* < 0.05).

The absolute PEs were calculated for all highly myopic eyes (Table 2). No significant differences were found in the MedAEs between the TK and standard K methods (Wilcoxon signed rank test, *P* > 0.05 in all pairs). However, though without statistical significance, the TK method showed slightly smaller SD values of PE than the standard K method using newer generation formulas, including XGBoost enhancement calculator (0.518 vs. 0.572), RBF 3.0 formula (0.527 vs. 0.536), Kane formula (0.536 vs. 0.539), and BUII formula (0.604 vs. 0.630).

**Table 2:** The absolute prediction errors of different formulas using total keratometry or standard keratometry in highly myopic eyes

IOL formula	SD of PE	TK method (n = 103)		*P*1 value for multiple comparisons	SD of PE	K method (n = 103)		*P*2 value for multiple comparisons
		MAE ± Std	MedAE			MAE ± Std	MedAE	
XGB	0.518	0.379 ± 0.351	0.324	–	0.572	0.409 ± 0.398	0.296	–
RBF 3.0	0.527	0.411 ± 0.341	0.340	–	0.536	0.419 ± 0.352	0.355	–
Kane	0.536	0.518 ± 0.365	0.435	*P*1 (vs. XGB) = 0.018*	0.539	0.527 ± 0.381	0.475	–
BUII	0.604	0.516 ± 0.401	0.435	*P*1 (vs. XGB) = 0.022*	0.630	0.536 ± 0.414	0.445	*P*2 (vs. XGB) = 0.021*
EVO 2.0	0.593	0.530 ± 0.400	0.440	*P*1 (vs. XGB) < 0.001* *P*1 (vs. RBF) = 0.002*	0.591	0.532 ± 0.412	0.425	*P*2 (vs. XGB) = 0.002* *P*2 (vs. RBF) = 0.005*
K6	0.737	0.579 ± 0.489	0.435	*P*1 (vs. XGB) = 0.017*	0.731	0.572 ± 0.487	0.415	–
Haigis^WK^	0.692	0.569 ± 0.465	0.460	*P*1 (vs. XGB) < 0.001* *P*1 (vs. RBF) = 0.003*	0.686	0.563 ± 0.471	0.465	*P*2 (vs. XGB) < 0.001* *P*2 (vs. RBF) = 0.002*
Haigis	0.712	0.589 ± 0.488	0.450	*P*1(vs. XGB) = 0.004* *P*1 (vs. RBF) = 0.020*	0.715	0.584 ± 0.483	0.475	*P*2(vs. XGB) = 0.016* *P*2(vs. RBF) = 0.040*
SRK/T^WK^	0.621	0.535 ± 0.401	0.495	*P*1 (vs. XGB) = 0.004* *P*1 (vs. RBF) = 0.017*	0.629	0.544 ± 0.409	0.465	*P*2(vs. XGB) = 0.003* *P*2(vs. RBF) = 0.009*
SRK/T	0.733	0.565 ± 0.470	0.460	*P*1 (vs. XGB) = 0.067*	0.720	0.550 ± 0.466	0.445	–

*TK* = total keratometry; *K* = standard keratometry; *SD* = standard deviation; *PE* = prediction error; *MAE* = mean absolute error; *Std* = standard deviation; *MedAE* = median absolute error; *XGB* = XGBoost enhancement calculator; *RBF 3.0* = Radial Basis Function formula version 3.0; *BUII* = Barrett Universal II formula; *EVO 2.0* = Emmetropia Verifying Optical formula version 2.0; *K6 *= Cooke K6 formula

*P*1: *P* value of the Bonferroni correction for multiple comparisons with all TK method

*P*2: *P* value of the Bonferroni correction for multiple comparisons with all K method

^*^Statistically different (*P* < 0.05)

Amongst all ten formulas, the XGBoost enhancement calculator, RBF 3.0 and Kane formula ranked top three when considering SD of PEs in both TK and K groups (H test, *P* < 0.05). Particularly, the XGBoost enhancement calculator TK method demonstrated the lowest MAE (0.379 ± 0.351 D). In the TK group, the MedAE of the XGBoost enhancement formula was not significantly different from RBF 3.0 formula but was significantly lower than the other eight formulas; while the MedAE of the RBF 3.0 formula was not different statistically from both XGBoost enhancement calculator and Kane formula but was significantly lower than the other seven formulas (all *P* < 0.05 with Bonferroni correction). In the standard K group, the MedAE of the XGBoost enhancement formula was not statistically different with RBF 3.0, Kane, K6, and SRK/T formulas, but was statistically lower than five other formulas; while the MedAE of the RBF 3.0 formula was not statistically different with XGBoost enhancement calculator, Kane, BUII, K6, and SRK/T formulas, but was significantly lower than three other formulas (all *P* < 0.05 with Bonferroni correction).

**Fig. 4:**
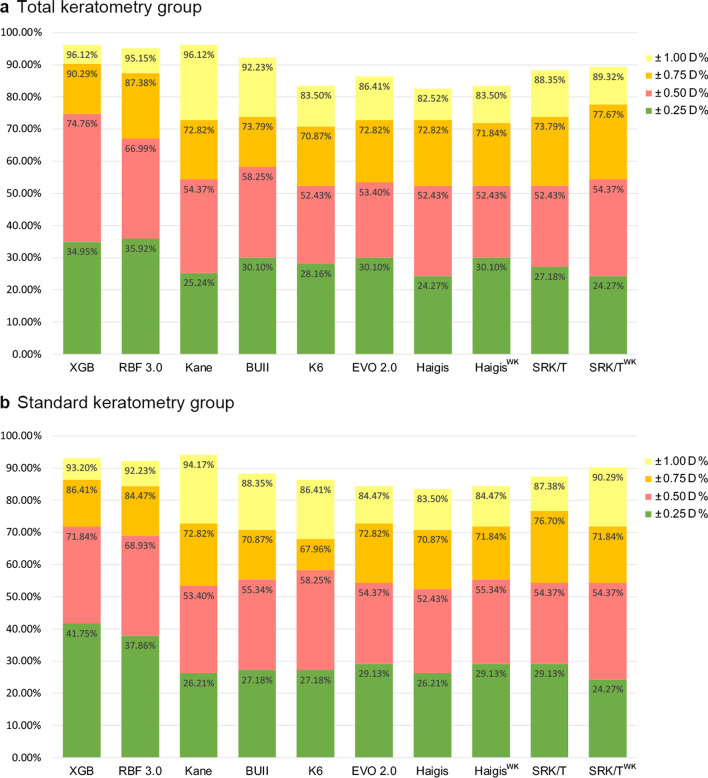
Percentages of eyes within ± 0.25 D, ± 0.50 D, ± 0.75 D and ± 1.00 D of prediction errors using different formulas with total keratometry (TK) and standard keratometry (K). XGB, XGBoost enhancement calculator; RBF 3.0, Radial Basis Function formula 3.0; BUII, Barrett Universal II formula; K6, Cooke K6 formula; EVO 2.0, Emmetropia Verifying Optical formula version 2.0

The percentages of eyes within ± 0.25 D, ± 0.50 D, ± 0.75 D and ± 1.00 D of the PE were further compared in both TK and K groups (Fig. 4). No statistical difference was found in ± 0.2 5D % and ± 1.00 D %. In the TK group, the XGBoost enhancement calculator showed comparable ± 0.50 D % with RBF 3.0 formula (74.76% vs. 66.99%, *P* > 0.05), and significantly larger ± 0.50 D % than eight other formulas (Cochran's Q test, adjusted *P* value < 0.05); it also showed comparable ± 0.75 D % with both RBF 3.0 and BUII formula (90.29% vs. 87.38% and 73.79%, respectively, both *P* > 0.05), and was statistically larger ± 0.75 D % than seven other formulas (Cochran's Q test, adjusted *P* value < 0.05). In the K group, the XGBoost enhancement calculator showed significantly larger ± 0.50 D % and ± 0.75 D % than Kane, EVO 2.0, and Haigis formulas (Cochran's Q test, adjusted *P* value < 0.05), and was not significantly different from six other formulas.

### Subgroup comparisons

A subgroup analysis was conducted (Additional file 1: Table S2), and no statistically significant differences were found between the TK and K methods for any subgroup (all *P* > 0.05). In the AL 26.00 to 28.00 mm group, the MedAE was significantly lower in XGboost compared to EVO 2.0 and Haigis^WK^ formulas in both TK and K groups. In the AL 28.00 to 30.00 mm group, the MedAE was statistically lower in RBF 3.0 formula compared to K6 formula in the TK group. In the AL > 30.00 mm group, the MedAE were statistically lower in XGboost, and RBF 3.0 compared to Kane, Haigis, and SRK/T in TK group, or compared to Kane and Haigis in K group (all *P* < 0.05 with Bonferroni correction).

## Discussion

Even with continuously advanced IOL power calculation formulas, cataract surgery on highly myopic eyes often results in unexpected refractive outcomes [12]. Recent improvements in TK biometry have demonstrated benefits in IOL calculation [16, 17, 27–29], but few studies have demonstrated its potential in highly myopic eyes, especially for those without prior refractive surgeries [13]. In this study, we demonstrated that highly myopic eyes with thinner CCTs tend to have larger differences between TK and K methods, while the XGBoost enhancement calculator and RBF 3.0 formula, with either TK or K method, seemed to be the most promising options for IOL power calculation for this special population.

The TK method concept is relatively new and its difference from standard K is worth investigating. We demonstrated relatively good agreement between the two methods in highly myopic eyes. Shajari et al. have also shown comparable astigmatism measurements between TK and standard K in normal AL eyes [30]. A greater difference between TK and K has previously been associated with flatter corneas in eyes that have undergone myopic refractive surgeries [27]. In this study, though there was good agreement, a larger difference between TK and K values was found in highly myopic eyes with thinner CCTs. It might be because the assessment of the posterior corneal surface with the IOLMaster 700 was done with consideration for the corneal pachymetry data. Therefore, for eyes with thinner corneal thicknesses, it might be that the TK measurement generates more accurate outcomes and should be recommended.

The improvement of K measurement in IOL power calculation began with the invention of ray tracing techniques using a rotating Scheimpflug camera [30]. Some studies have revealed that though Pentacam K readings (such as true net power and total corneal refractive power) differ from IOLMaster standard K readings, the PEs obtained with each machine are comparable for normal eyes [31–33]. The newer IOLMaster 700 obtains a reading of total corneal power, taking both corneal thickness and actual values for the radius of the posterior cornea into account, using telecentric 3-zone K and swept-source OCT technology. By replacing hypotheses and modeling with actual measurements, the IOLMaster 700 may provide reliable data on corneal power in some challenging cases, such as surgically modified [7, 28, 29, 34] and high astigmatic corneas [17, 35, 36]. Here, we found no significant difference between TK and standard K in highly myopic eyes within this certain range of corneal thickness. Tsessler et al. also found that the use of TK did not provide significant improvement to its prediction accuracy for all AL ranges [13]. However, Fabian et al. concluded that TK was better than standard K for normal eyes [17]. This can be attributed to the study population which included only astigmatic eyes (K ≥ 0.75 D) [17]. Here, in order to determine the agreement and fitness of TK in highly myopic eyes with a wide range of CCTs, we defined no restrictions on corneal astigmatism and even excluded highly astigmatic eyes needing Toric IOL implantations. Still, more attention on TK implementation should be paid to highly myopic eyes with thinner corneas. In addition, using intraoperative aberrometry to measure aphakic refraction was demonstrated to return more accurate results than preoperative biometry using standard K values for highly myopic eyes [37]. The accuracy comparison of intraoperative aberrometry and the new TK measurement may merit further investigation.

Many efforts have been made to determine the most accurate formula for highly myopic eyes [2, 12, 22, 38]. Intentional myopic overcorrection is typically planned for cataract surgeries on highly myopic patients [38], so that they may continue living with their familiar short-sightedness. Here, though most of the MedAEs and MAEs were relatively lower with the TK method than the K method, no significant difference between the two methods was observed. After applying the TK value, we still found different prediction accuracies for the ten formulas. The most promising in all highly myopic eyes might be two of the three AI formulas, the XGBoost enhancement calculator and RBF 3.0 formula. The XGBoost enhancement calculator was specifically designed to optimize the refractive prediction for highly myopic eyes using machine learning [14]. The RBF 3.0 formula, an improvement from the 2.0 version, employed pattern recognition and a sophisticated form of data interpolation [13]. The RBF 3.0 formula only accepts target refraction values from + 1.0 D to − 2.5 D. Therefore, the XGBoost enhancement calculator might be more useful when less than − 2.5 D myopic refractive targets were scheduled for extremely long eyes. The TK value seemed to fit the newer AI-based and traditional vergence formulas, but further optimization might be achieved by using more real-world TK measurements and prediction outcomes.

Although the separation into subgroups might lower the power for paired comparisons of TK and K methods, it is still interesting to note that K method for the 28–30 mm subgroup (as compared to the TK group) had lower MedAEs in nine formulas (XGBoost, RBF, Kane, BUII, EVO, K6, Haigis^WK^, SRK/T, and SRK/T^WK^). However, for the AL > 30 mm subgroup, the TK methods showed lower MedAEs for XGBoost, RBF and Kane, but not the others. These suggest that for eyes with AL > 30 mm, using new TK method with these fourth-generation formulas has more potential in terms of accuracy. Encouragingly, promising outcomes were still revealed when comparing performance of ten formulas in a subgroup analysis. Particularly, the XGBoost and RBF 3.0 formula seemed to work much better for eyes with AL > 30 mm, demonstrating lower MedAEs than the other eight formulas.

The limitation of this study would be that the formula comparison results are applicable to this model of IOL (HumanOptics MC X11 ASP). Since other IOL models have different geometries or optical zones, future studies will be needed to determine that these results are repeatable in other IOL models.

## Conclusions

In conclusion, for highly myopic eyes, the TK method showed good agreement with the standard K, yet a larger difference between the TK and K methods was found in highly myopic eyes with thinner corneas. We also verified that TK can be incorporated into modern IOL power calculations, while the XGBoost enhancement calculator and RBF 3.0, using either the TK or K method, showed more significantly promising outcomes than other formulas in highly myopic eyes.

## Supplementary Information

**Additional file 1: Table S1. **Agreement between total keratometry and standard keratometry in different central corneal thickness subgroups**. Table S2.** Absolute prediction errors of different IOL formulas using total keratometry or standard keratometry in highly myopic subgroups**.**

## Data Availability

The data that support the findings of this study are available from the corresponding author upon reasonable request.
