# Supplementary material for: Application of total keratometry in ten intraocular lens power calculation formulas in highly myopic eyes
Source: Eye Vis (Lond). 2022 Jun 9;9:21. doi: 10.1186/s40662-022-00293-3 (PMC9178866; doi:10.1186/s40662-022-00293-3)
Supplement: Supplementary file 1 — Additional file 1: Table S1. Agreement between total keratometry and standard keratometry in different central corneal thickness subgroups. Table S2. Absolute prediction errors of different IOL formulas using total keratometry or standard keratometry in highly myopic subgroups. [file 40662_2022_293_MOESM1_ESM.docx]

**Table S1.** Agreement between total keratometry and standard keratometry in different central corneal thickness subgroups.

| CCT subgroup | < 490 μm | 490 to 560 μm | 560 to 600 μm | > 600 μm |
| --- | --- | --- | --- | --- |
| n | 6 | 58 | 32 | 7 |
| K flat and TK flat | |  |  |  |
| ICC | 0.997 | 0.994 | 0.995 | 0.997 |
| *P* value | <0.001 | <0.001 | <0.001 | <0.001 |
| K steep and TK steep | |  |  |  |
| ICC | 0.998 | 0.995 | 0.995 | 0.999 |
| *P* value | <0.001 | <0.001 | <0.001 | <0.001 |
| K and TK | | |  |  |
| ICC | 0.998 | 0.997 | 0.998 | 1.000 |
| *P* value | <0.001 | <0.001 | <0.001 | <0.001 |

*CCT* = central corneal thickness; *K* = standard keratometry; *TK* = total keratometry; *ICC* = intraclass correlation coefficient

**Table S2.** Absolute prediction errors of different IOL formulas using total keratometry or standard keratometry in highly myopic subgroups.

| IOL formula |  | TK method (n=103) | | *P*1 value for multiple comparisons |  | K method (n=103) | | *P*2 value for multiple comparisons |  |  |  |  |
| --- | --- | --- | --- | --- | --- | --- | --- | --- | --- | --- | --- | --- |
|  | SD of PE | MAE ± Std | MedAE |  | SD of PE | MAE ± Std | MedAE |  |  |  |  |  |
| AL 26.00 to 28.00 mm (n=46) | | | | |  |  |  |  |  |  |  |  |
| XGB | 0.464 | 0.381 ± 0.264 | 0.340 | - | 0.530 | 0.418 ± 0.33 | 0.409 | - |  |  |  |  |
| RBF 3.0 | 0.524 | 0.48 ± 0.309 | 0.413 | - | 0.499 | 0.463 ± 0.313 | 0.375 | - |  |  |  |  |
| Kane | 0.451 | 0.479 ± 0.331 | 0.428 | - | 0.453 | 0.48 ± 0.348 | 0.470 | - |  |  |  |  |
| BUII | 0.466 | 0.515 ± 0.356 | 0.425 | - | 0.509 | 0.549 ± 0.374 | 0.470 | - |  |  |  |  |
| EVO 2.0 | 0.461 | 0.582 ± 0.391 | 0.550 | *P*1(*vs.* XGB)<0.001* | 0.475 | 0.593 ± 0.403 | 0.535 | *P*2(*vs.* XGB)<0.001* |  |  |  |  |
| K6 | 0.726 | 0.668 ± 0.473 | 0.615 | - | 0.729 | 0.651 ± 0.493 | 0.613 | - |  |  |  |  |
| Haigis^WK^ | 0.588 | 0.604 ± 0.454 | 0.488 | *P*1(*vs.* XGB)=0.002* | 0.606 | 0.628 ± 0.46 | 0.518 | *P*2(*vs.* XGB)<0.001*  *P*2(*vs.* Kane)=0.038* |  |  |  |  |
| Haigis | 0.602 | 0.474 ± 0.365 | 0.400 | *P*1(*vs.* EVO)=0.035* | 0.631 | 0.501 ± 0.377 | 0.405 | - |  |  |  |  |
| SRK/T^WK^ | 0.545 | 0.53 ± 0.327 | 0.515 | - | 0.555 | 0.542 ± 0.324 | 0.470 | - |  |  |  |  |
| SRK/T | 0.536 | 0.505 ± 0.322 | 0.470 | - | 0.547 | 0.516 ± 0.335 | 0.475 | - |  |  |  |  |
| AL 28.00 to 30.00 mm (n=29) | | | | |  |  |  |  |  |  |  | - |
| XGB | 0.704 | 0.453 ± 0.537 | 0.329 | - | 0.773 | 0.5 ± 0.586 | 0.286 | - |  |  |  |  |
| RBF 3.0 | 0.626 | 0.414 ± 0.463 | 0.265 | - | 0.674 | 0.448 ± 0.498 | 0.225 | - |  |  |  |  |
| Kane | 0.678 | 0.533 ± 0.477 | 0.405 | - | 0.681 | 0.533 ± 0.501 | 0.315 | - |  |  |  |  |
| BUII | 0.723 | 0.561 ± 0.524 | 0.445 | - | 0.764 | 0.584 ± 0.543 | 0.440 | - |  |  |  |  |
| EVO 2.0 | 0.680 | 0.547 ± 0.466 | 0.405 | - | 0.668 | 0.54 ± 0.478 | 0.390 | - |  |  |  |  |
| K6 | 0.838 | 0.613 ± 0.581 | 0.445 | *P*1(*vs.* RBF)=0.041* | 0.830 | 0.603 ± 0.576 | 0.410 | - |  |  |  |  |
| Haigis^WK^ | 0.779 | 0.598 ± 0.536 | 0.485 | - | 0.765 | 0.575 ± 0.554 | 0.410 | - |  |  |  |  |
| Haigis | 0.795 | 0.637 ± 0.568 | 0.450 | - | 0.782 | 0.612 ± 0.56 | 0.470 | - |  |  |  |  |
| SRK/T^WK^ | 0.707 | 0.554 ± 0.516 | 0.470 | - | 0.712 | 0.553 ± 0.528 | 0.410 | - |  |  |  |  |
| SRK/T | 0.707 | 0.504 ± 0.499 | 0.455 | - | 0.675 | 0.485 ± 0.468 | 0.410 | - |  |  |  |  |
| AL >30.00 mm (n=28) | | | | |  |  |  |  |  |  |  |  |
| XGB | 0.353 | 0.299 ± 0.195 | 0.236 | - | 0.358 | 0.299 ± 0.2 | 0.244 | - |  |  |  |  |
| RBF 3.0 | 0.347 | 0.294 ± 0.189 | 0.258 | - | 0.367 | 0.316 ± 0.179 | 0.340 | - |  |  |  |  |
| Kane | 0.507 | 0.567 ± 0.281 | 0.570 | *P*1(*vs.* XGB)=0.024*  *P*1(*vs.* RBF)=0.008* | 0.514 | 0.599 ± 0.276 | 0.603 | *P*2(*vs.* XGB)=0.004*  *P*2(*vs.* RBF)=0.008* |  |  |  |  |
| BUII | 0.581 | 0.474 ± 0.327 | 0.433 | - | 0.570 | 0.465 ± 0.318 | 0.398 | - |  |  |  |  |
| EVO 2.0 | 0.543 | 0.425 ± 0.33 | 0.395 | - | 0.547 | 0.423 ± 0.338 | 0.333 | - |  |  |  |  |
| K6 | 0.526 | 0.396 ± 0.361 | 0.265 | - | 0.523 | 0.411 ± 0.327 | 0.290 | - |  |  |  |  |
| Haigis^WK^ | 0.631 | 0.482 ± 0.405 | 0.433 | - | 0.583 | 0.443 ± 0.38 | 0.393 | - |  |  |  |  |
| Haigis | 0.600 | 0.727 ± 0.548 | 0.603 | *P*1(*vs.* XGB)=0.003*  *P*1(*vs.* RBF)<0.001*  *P*1(*vs.* K6)=0.031* | 0.553 | 0.691 ± 0.543 | 0.588 | *P*2(*vs.* XGB)=0.005*  *P*2(*vs.* RBF)=0.002* |  |  |  |  |
| SRK/T^WK^ | 0.648 | 0.522 ± 0.391 | 0.475 | - | 0.664 | 0.538 ± 0.409 | 0.418 | - |  |  |  |  |
| SRK/T | 0.708 | 0.729 ± 0.606 | 0.588 | *P*1(*vs.* XGB)=0.006*  *P*1(*vs.* RBF)=0.002* | 0.661 | 0.675 ± 0.62 | 0.528 | - |  |  |  |  |

TK = total keratometry; K = standard keratometry; SD = standard deviation; PE = prediction error; MAE = mean absolute error; Std = standard deviation; MedAE = median absolute error; XGB = XGBoost enhancement calculator; RBF 3.0 = Radial Basis Function formula version 3.0; BUII = Barrett Universal II formula; EVO 2.0 = Emmetropia Verifying Optical formula version 2.0; K6 = Cooke K6 formula

*P*1: *P* value of the Bonferroni correction for multiple comparisons with all TK method.

*P*2: *P* value of the Bonferroni correction for multiple comparisons with all K method.

*Statistically different (*P* <0.05)
